# Nucleosomal asymmetry: a novel mechanism to regulate nucleosome function

**DOI:** 10.1042/BST20230877

**Published:** 2024-05-23

**Authors:** Devisree Valsakumar, Philipp Voigt

**Affiliations:** 1 Epigenetics Programme, Babraham Institute, Cambridge CB22 3AT, U.K.; 2 Wellcome Centre for Cell Biology, School of Biological Sciences, University of Edinburgh, Edinburgh EH9 3BF, U.K.

**Keywords:** bivalent domains, chromatin, histone modifications, histones, nucleosomes, transcription

## Abstract

Nucleosomes constitute the fundamental building blocks of chromatin. They are comprised of DNA wrapped around a histone octamer formed of two copies each of the four core histones H2A, H2B, H3, and H4. Nucleosomal histones undergo a plethora of posttranslational modifications that regulate gene expression and other chromatin-templated processes by altering chromatin structure or by recruiting effector proteins. Given their symmetric arrangement, the sister histones within a nucleosome have commonly been considered to be equivalent and to carry the same modifications. However, it is now clear that nucleosomes can exhibit asymmetry, combining differentially modified sister histones or different variants of the same histone within a single nucleosome. Enabled by the development of novel tools that allow generating asymmetrically modified nucleosomes, recent biochemical and cell-based studies have begun to shed light on the origins and functional consequences of nucleosomal asymmetry. These studies indicate that nucleosomal asymmetry represents a novel regulatory mechanism in the establishment and functional readout of chromatin states. Asymmetry expands the combinatorial space available for setting up complex sets of histone marks at individual nucleosomes, regulating multivalent interactions with histone modifiers and readers. The resulting functional consequences of asymmetry regulate transcription, poising of developmental gene expression by bivalent chromatin, and the mechanisms by which oncohistones deregulate chromatin states in cancer. Here, we review recent progress and current challenges in uncovering the mechanisms and biological functions of nucleosomal asymmetry.

## Introduction

Nucleosomes are the basic building blocks of chromatin. They are comprised of 147 bp of DNA wrapped around a histone octamer that is formed of two copies each of the four core histones H2A, H2B, H3, and H4 [1,2]. By regulating access to genomic DNA, nucleosomes are at the center of virtually all chromatin-templated processes, controlling transcription, replication, and DNA repair. Histones within nucleosomes are subject to a wide range of site-specific posttranslational modifications, including methylation, acetylation, phosphorylation, and ubiquitination [3,4]. Moreover, the canonical core histones can be replaced with histone variants, providing additional means to regulate nucleosome structure and function [5,6]. Together, histone marks and histone variants set up chromatin states that reflect and direct events such as transcriptional activation and repression by directly altering chromatin structure or by recruiting specific binding or ‘reader’ proteins [3,7,8].

Since David Allis and colleagues coined the ‘histone code’ hypothesis in the early 2000s [9,10], it has become increasingly clear that histone marks commonly function in a combinatorial fashion. This is supported by genome-wide studies that have revealed co-occurrence of different histone marks and variants at specific genomic regions such as active promoters and poised enhancers [8,11,12]. At the same time, biochemical and structural studies have provided insight into the mechanisms that underlie synergistic and antagonistic relationships between different histone marks [13–15]. Many proteins that interact with chromatin comprise multiple binding domains that allow them to interact not only with combinations of different histone marks, but also with other chromatin features such as DNA or the nucleosome acidic patch. Such multivalent engagement governs the affinity, dynamics, and specificity of interactions in chromatin, rendering multivalency a key regulatory principle in chromatin biology [16–19].

As nucleosomes contain two copies each of the four core histones, each sister histone could theoretically carry different marks, allowing them to act independently rather than redundantly in mediating multivalent interactions with combinatorial histone codes. However, given that sister histones within nucleosomes are related through 2-fold rotational pseudo symmetry, they have for a long time been considered to be identical and to therefore carry the same modifications. However, when this assumption was eventually put to a test, mass spectrometry and single molecule imaging-based approaches revealed that nucleosomes in mouse and human cells can in fact be asymmetrically modified, carrying different marks on equivalent sister histones [20,21]. Rather than being a rare event, nucleosomal asymmetry was found to be pervasive, with about half of all nucleosomes modified with H3K27me2/3 or H4K20me1 carrying the mark on only one of the two H3 or H4 sister histones, respectively [21]. Moreover, studies based on high-resolution chromatin immunoprecipitation coupled to exonuclease treatment (ChIP-exo) showed that nucleosomes at the transcription start sites (TSS) of about half of all genes in budding yeast similarly display asymmetry [22]. In addition to uncovering the existence of asymmetric nucleosomes, these pioneering studies also provided first insights into the mechanisms that give rise to asymmetry and identified transcription and the establishment and function of bivalent chromatin as key cellular processes that are regulated by nucleosomal asymmetry. Since then, a growing number of studies have corroborated the notion that nucleosomal asymmetry represents a new layer of regulation in the establishment, readout, and potentially inheritance of combinatorial histone codes and chromatin states, opening an exciting new area of chromatin biology.

Here, we will review recent progress and current challenges in elucidating the origins and functional consequences of nucleosomal asymmetry. We will summarize our current understanding of how asymmetry arises, how it affects the function of chromatin remodelers and histone mark readers, and how it regulates cellular processes such as transcription. We will also highlight some of the methods developed to study nucleosomal asymmetry *in vitro* and *in vivo*.

## Origins of nucleosomal asymmetry

**Figure 1.**
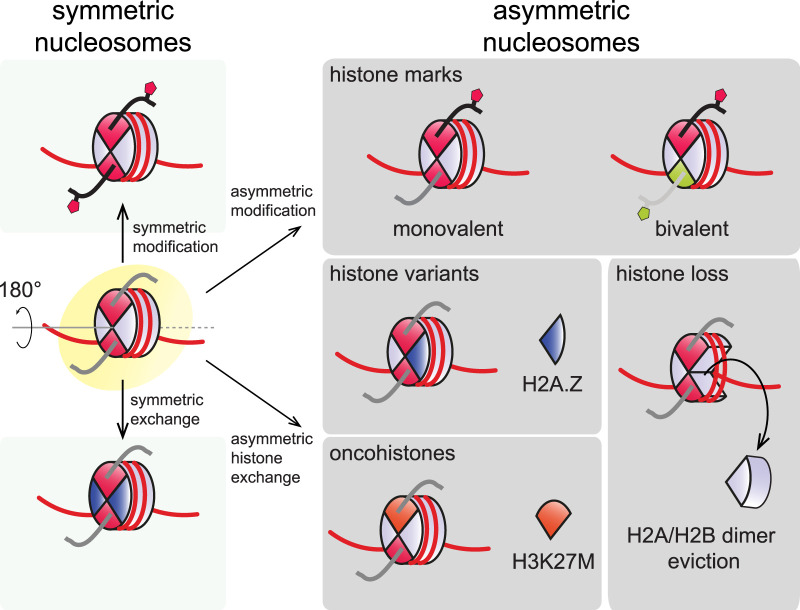
Flavors of nucleosomal asymmetry. Sister histones within nucleosomes are related through two-fold rotational pseudo symmetry. In symmetric nucleosomes, sister histones carry identical modifications or are replaced by the same histone variants. This symmetry is broken in asymmetric nucleosomes. Only one of two sister histones can carry a specific modification, or each sister histone can carry a different mark to form a bivalent asymmetric nucleosome. Similarly, histone variants or oncohistones can be incorporated in an asymmetric fashion. H2A/H2B dimers can also be lost from nucleosomes asymmetrically, forming hexasomes.

Nucleosomal asymmetry manifests in several ways (Figure 1). Besides sister histones exhibiting distinct modifications, histone isoforms can be incorporated into nucleosomes asymmetrically, e.g. by pairing canonical histone H2A with the histone variant H2A.Z in a heterotypic nucleosome [22–24]. Nucleosomes may also lack an H2A-H2B dimer altogether, forming so-called hexasomes [22]. Mutations in histone genes have been found in various cancers [25–29]. As these mutations only affect one of multiple copies encoding the same core histone, the abundance of these ‘oncohistones’ is low compared with their wild-type counterparts, resulting in predominantly asymmetric incorporation into nucleosomes (see e.g. [30,31]). The nature of these types of asymmetry suggest that histone modifying enzymes, chromatin remodelers, and histone chaperones are responsible for the formation of asymmetric nucleosomes. These enzymes may generate asymmetry *de novo* as a consequence of their intrinsic catalytic properties, or they may recognize and propagate asymmetry that already exists within a nucleosome or its environment. Such pre-existing asymmetry may involve other histone marks and variants as well as the underlying DNA sequence. As we will discuss below, both modes of regulation appear to be at play *in vivo*.

### Properties of histone modifiers that give rise to asymmetry

In the absence of any pre-existing asymmetry, nucleosomal asymmetry of histone marks may arise stochastically due to incomplete substrate turnover by histone modifying enzymes. In the case of histone marks, modifiers may be prevented from catalyzing symmetric modifications by limited enzyme availability, poor catalytic activity, or weak interaction with nucleosome substrates, resulting in residence times that are too short to ensure full substrate conversion. In support of such a scenario, mass spectrometry-based analysis of nucleosomes that were affinity-purified using antibodies against specific histone marks revealed that substantial amounts of nucleosomes are asymmetrically modified with respect to the marks tested [21]. Similarly, calibrated ChIP-seq studies with internal standards that allow to convert ChIP enrichments to biologically meaningful, normalized modification densities showed that histone marks rarely reach maximal modification density across the genome outside of specific loci [32]. Poor substrate turnover may therefore represent a simple, general mechanism that gives rise to nucleosomal asymmetry genome-wide. However, it remains to be determined exactly how these biochemical properties interface with other dynamic processes that take place in the complex environment of native chromatin, including nucleosome turnover, competition between different modifiers, and the action of eraser enzymes such as demethylases.

Several histone modifying enzymes exhibit loose substrate specificity and could thus establish asymmetry by stochastically modifying different residues on sister histones within a nucleosome. This has been demonstrated biochemically for checkpoint kinase 1 (CHK1) and protein kinase C (PKC) family enzymes [33]. CHK1 phosphorylates both H3S10 and H3T11 with similar efficiency, albeit in a mutually exclusive fashion, leading to generation of asymmetric nucleosomes. Similarly, PKCα and PKCβ phosphorylate either H3T6 or H3S10 in a mutually exclusive fashion and therefore also give rise to asymmetry *in vitro* [33]. Beyond kinases, many histone acetyltransferases (HATs) are also known to have broad substrate specificity, targeting several lysine residues within the same histone. For instance, p300 and its homolog CBP both target H3K18 and H3K27, among other substrates [34]. Mutually exclusive acetylation of residues on histone H3 would give rise to asymmetric acetylation. Even though histone methyltransferases usually only target a single specific histone residue, incomplete turnover may also lead to asymmetry through combination of different mono-, di- or trimethylation states across sister histones. Taken together, the *de novo* establishment of nucleosomal asymmetry may be a consequence of the catalytic properties of histone modifying enzymes. However, it remains to be determined how strongly these processes contribute to setting up asymmetry *in vivo* in the context of the complex chromatin environment within the cell nucleus, where site-specific recruitment of factors will lead to varying local abundance and residence times of specific enzymes. Moreover, such an environment will involve competition between different histone modifiers, leading to asymmetry via cross-talk between different marks, enzymes, and readers as discussed below.

In the case of heterotypic nucleosomes, similar mechanisms based on incomplete turnover may be responsible for the asymmetric incorporation of histone variants such as H2A.Z. The yeast SWR1C chromatin remodeling complex and its mammalian homolog SRCAP are responsible for replacing H2A-H2B dimers with H2A.Z-H2B dimers in nucleosomes [35]. Interestingly, the SWR1C complex catalyzes dimer exchange in a sequential, stepwise fashion, first exchanging one and then the other dimer. Kinetic analyses revealed that the second exchange reaction is markedly slower [36], thus promoting incomplete, asymmetric dimer exchange. The exchange reaction is further modulated by the DNA sequence surrounding the nucleosome substrate. At the TSS, SWR tends to bind on the side of the +1 nucleosome that borders the nucleosome free region (NFR), due to its preferential binding around long DNA linkers [37]. Moreover, runs of GC base pairs stimulate SWR activity and impart directionality onto dimer exchange, as H2A.Z-H2B incorporation is more efficient when the outgoing H2A-H2B dimer contacts DNA with higher GC content [38]. These findings suggest that nucleosomal DNA sequence plays an important role in directing H2A.Z to the distal side of the +1 nucleosome in an asymmetric fashion, as is observed in the yeast genome [22] (see also below). Indeed, the proximal and distal halves of nucleosomal DNA exhibit differences in base composition that render those nucleosomes asymmetric [39]. In addition to SWR, other chromatin remodelers such as Chd1 [40] and INO80 [41] exhibit sequences preferences in their binding and activity, giving directionality to nucleosome repositioning and downstream processes such as transcription. Moreover, transcription is an inherently asymmetric process, proceeding through a gene from its TSS to the transcription termination site in a directional fashion. While DNA sequence properties promote directionality of transcription, transcription itself likely further reinforces asymmetry and thus plays a key role in the generation of heterotypic nucleosomes and asymmetrically modified nucleosomes around the TSS and over the gene body.

Beyond chromatin remodelers, inherent properties of DNA sequences might also regulate the activity of histone modifying enzymes. Differences in DNA sequence and base composition have been shown to differentially regulate DNA shape and rigidity as well as the strength of histone-DNA contacts across the two nucleosome halves and their adjacent linkers [41–44], providing an asymmetric environment for modifiers to act in. However, the mechanisms and physiological consequences of such DNA sequence and structure-based regulation of histone mark asymmetry remain largely unexplored so far.

### Mechanisms promoting symmetric modification

**Figure 2.**
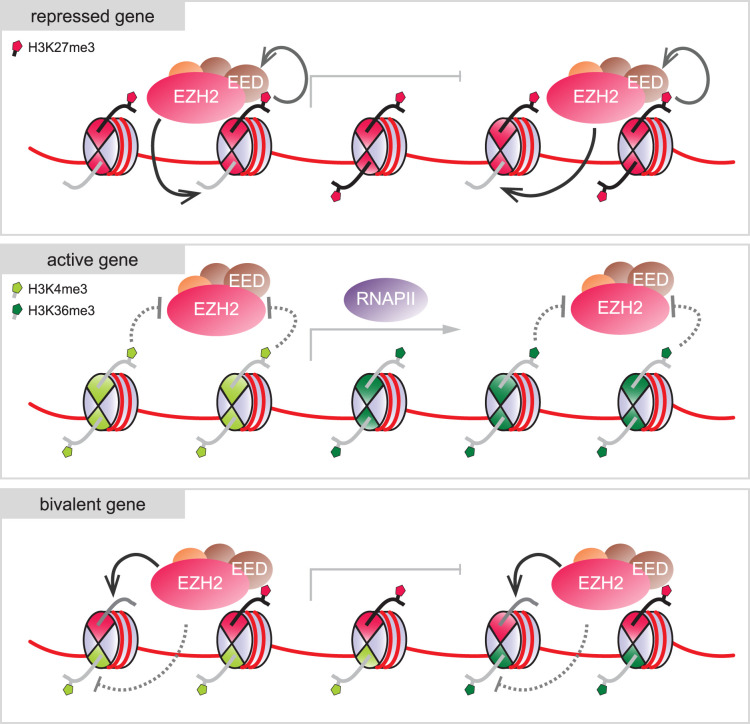
Feedback loops and histone mark cross-talk regulate symmetry, asymmetry, and bivalency. The activity of Polycomb repressive complex 2 (PRC2) and the symmetry state of its mark H3K27me3 is regulated by feedback loops and histone mark cross-talk. In repressed genes, a feedback loop stimulates symmetric modification. The EED subunit of PRC2 binds its catalytic product H3K27me3, thereby allosterically activating the catalytic subunit EZH2 (or its homolog EZH1) and promoting symmetric modification. In active genes, symmetric H3K4me3 and H3K36me3 marks prevent catalytic activity of PRC2, ensuring that promoters and gene bodies remain free of repressive H3K27me3. At bivalent promoters, asymmetric presence of H3K4me3 permits PRC2-mediated H3K27 trimethylation of the unmodified H3 sister histone, as inhibition only occurs if H3K4me3 is present on the same histone tail in cis. Analogous mechanisms allow the formation of nucleosomes carrying both H3K27me3 and H3K36me3 in asymmetric fashion.

Interestingly, many histone modifying enzymes exhibit properties that promote symmetric modification. Methyltransferase and acetyltransferase complexes often contain binding domains that recognize the mark placed by the enzyme, establishing autoregulatory positive feedback loops that promote recruitment and/or catalytic activity of the enzyme. These mechanisms may also operate in an intra-nucleosomal, trans-tail fashion. The SAGA HAT complex primarily acetylates histone H3 at lysine 14 but also targets lysines 9, 18, and 23 [45,46]. The complex comprises a bromodomain that interacts with acetylated lysines, including the catalytic products of the SAGA complex itself. Interaction of the bromodomain with one acetylated histone H3 tail within asymmetric nucleosomes stimulates activity of the yeast SAGA complex towards the unmodified sister histone tail [47,48], promoting symmetric modification. As another example, Polycomb repressive complex 2 (PRC2) catalyzes the trimethylation of histone H3 lysine 27 (H3K27me3), a mark associated with promoters and gene bodies of repressed genes [49–51]. The EED subunit contains a WD40 domain that binds H3K27me3, thereby stimulating PRC2 methylation activity [52]. This stimulation also occurs in trans within asymmetric nucleosomes carrying one H3K27me3 mark [21,53,54], thereby boosting symmetric modification (Figure 2).

In the case of the yeast Set1 complex, such positive feedback via complex-intrinsic reader domains appears to work in concert with asymmetry-specific demethylation to ensure symmetric modification in chromatin. Set1 catalyzes H3K4me3, a hallmark of actively transcribed promoters in yeast and higher eukaryotes [55,56]. Its Spp1 subunit (CFP1 in mammals) binds to H3K4me3 via a PHD finger [57,58], which may result in cooperative methylation of sister histones as observed for PRC2. As an additional mechanism promoting symmetric modification, the Set1 complex forms a dimer in yeast, mediated by its Sdc1 subunit (DPY30 in mammals) [59]. Mutations in Sdc1 that disrupt dimerization virtually abolish H3K4me3 and strongly reduce H3K4me2 levels, both products of Set1 [59]. At the same time, the yeast H3K4me2/3 demethylase Jhd2 appears to specifically remove asymmetric H3K4me3 [59]. Mechanistically, this asymmetry-specific demethylation is mediated by a PHD finger in the demethylase that binds unmodified H3K4 and promotes interaction with nucleosomes containing asymmetric H3K4me3 [60–63]. Together, these findings suggest that methylation and demethylation activities act in concert to focus symmetric H3K4me3 on promoter nucleosomes, while removing spurious asymmetric H3K4me3 present elsewhere. Given the high degree of conservation of the enzymes involved, similar mechanisms may be at play in mammalian systems. Interestingly, PRC2 has also been shown to dimerize [64–66], suggesting that enzyme dimerization-based mechanisms may also be at play in other modification systems.

### Histone mark cross-talk as a source of nucleosomal asymmetry

Many histone modifying enzymes have been shown to be stimulated or inhibited by other histone marks, establishing cross-talk between different modification systems [15]. In contrast with autoregulatory feedback loops that promote symmetry, cross-talk between different histone marks may be key to establishing and reinforcing nucleosomal asymmetry. While H3K27me3 is associated with promoters and gene bodies of repressed genes, H3K4me3 and H3K36me3 mark promoters and gene bodies, respectively, of actively expressed genes [8,11]. PRC2 has been shown to be inhibited by H3K4me3 and H3K36me3 [67], explaining how repressive H3K27me3 is excluded from actively transcribed genes (Figure 2). However, together with the failure to detect histone H3 tails carrying both H3K4me3 and H3K27me3 *in vivo*, these findings challenged the existence of promoters marked with both H3K4me3 and H3K27me3. These so-called bivalent domains are thought to regulate expression of developmentally regulated genes in undifferentiated cells such as embryonic stem cells (ESCs), keeping them repressed in ESCs but poised for activation upon appropriate differentiation signals [68–72]. Resolving this conundrum, bivalent nucleosomes in ESC were found to be in an asymmetric configuration, carrying H3K4me3 and H3K27me3 on separate — rather than the same — histone H3 tails [20,21]. Moreover, using recombinant symmetrically and asymmetrically modified nucleosomes, inhibition of PRC2 by H3K4me3 and H3K36me3 was found to only occur within the same histone tail in cis [21,53,54]. When H3K4me3 or H3K36me3 were present asymmetrically on only one H3 sister histone within a nucleosome, PRC2 activity towards the unmodified histone H3 copy was unaffected, giving rise to an asymmetric bivalent nucleosome (Figure 2). These findings provided not only conclusive evidence for the existence of bivalent nucleosomes but also a mechanism for their generation.

Histone mark cross-talk has been found to generate asymmetry in other modification systems as well, such as H3K4 methylation-based inhibition of H3T3 phosphorylation by Haspin kinase [73]. Together, these findings highlight how histone mark cross-talk can establish asymmetric nucleosome states, thereby expanding the combinatorial space available for complex sets of histone marks at individual nucleosomes within chromatin. Cross-talk between mutually exclusive marks is a common feature of many histone modification systems and may therefore lead to asymmetry for many marks beyond the examples known so far.

## Approaches to generate asymmetrically modified nucleosomes for *in vitro* studies

**Figure 3.**
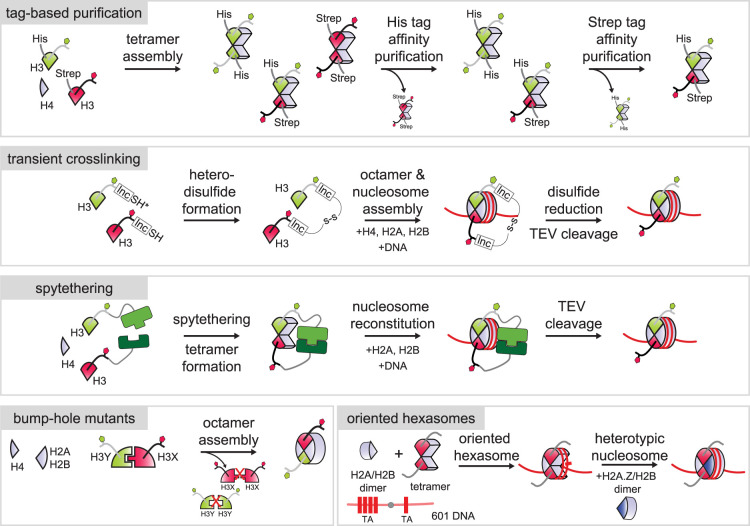
Tools to create asymmetric nucleosomes. Schematics outlining methods available for the generation of asymmetric nucleosomes *in vitro*. To generate nucleosomes carrying asymmetric modification on histone H3 or H4, approaches based on affinity tags or on transient cross-linking of asymmetric histone pairs can be employed. Spytethering similarly exploits joining the desired asymmetric histone combination together during octamer formation and nucleosome reconstitution, before traceless removal of the SpyCatcher and SpyTag fusion domain using TEV protease. Bump-hole mutation approaches have been devised to generate asymmetric nucleosomes carrying differentially modified forms of histone H3 or different variants or mutants of histone H3. They do not necessitate removal of tags after assembly. Hexasomes and heterotypic nucleosomes with different isoforms or mutants of H2A or H2B can be generated using directed hexasome assembly. This can involve truncated forms of the 601 nucleosome positioning DNA to promote formation of hexasomes and reduce contamination with nucleosome byproducts (not shown here).

The ability to generate recombinant nucleosomes that carry defined modifications in an asymmetric fashion was essential to uncovering the intra- but not inter-histone tail inhibition of PRC2 by active histone marks [21]. Initial protocols utilized affinity tags to generate asymmetric nucleosomes for biochemical studies. Each differentially modified sister histone was fused to an orthogonal affinity tag, allowing for sequential affinity purification to separate asymmetric histone octamers from symmetric byproducts of octamer reconstitution (Figure 3) [21]. These early approaches were laborious and characterized by a low yield of the desired asymmetric nucleosomes. Since these early studies, several groups have developed elegant novel approaches to generate asymmetrically modified nucleosomes (reviewed in-depth in [74]). As the first step, these approaches rely on native chemical ligation and other techniques to generate histones carrying specific modifications [75–77], which are then assembled into asymmetrically modified nucleosomes. These developments allow generating a plethora of asymmetric nucleosomes with ease, greatly facilitating determining the mechanisms that control the origins and functional consequences of nucleosomal asymmetry.

The Fierz group developed an approach based on joining the H3 sister histones in the desired asymmetric combination via a cleavable linker prior to octamer assembly, allowing for efficient generation of asymmetric octamers and subsequent traceless removal of the linker using TEV protease [53,54]. In that way, these approaches also eliminate any potential negative impact arising from the presence of affinity tags during downstream applications. This approach confirmed that PRC2 is inhibited by H3K4m3 and H3K36me3 only in cis, whereas H3K27me3 can stimulate PRC2 activity in trans in asymmetric nucleosomes [53,54]. The Muir group developed conceptually similar approaches for traceless generation of asymmetric histone octamers, most recently by employing the SpyCatcher/SpyTag protein heterodimerization system [63]. Using this spytethering-based approach, they provided direct biochemical evidence for the preferential demethylation of asymmetric H3K4me3 nucleosomes by the H3K3me3 demethylase KDM5B [63], as suggested for its Jhd2 homologue in yeast. Although other techniques are already available to generate nucleosomes with asymmetrically modified H2A or H2B (see next section), the SpyCatcher/SpyTag approach should be applicable to all four core histones.

Other approaches developed to generate asymmetric nucleosomes target the dimerization interface of the two H3 sister histones within the histone octamer. Using protein design followed by directed evolution in yeast, the Kaufman and Rando labs developed a pair of mutant H3 histones termed H3X and H3Y that efficiently heterodimerize but do not homodimerize, due to bump-hole mutations in the H3-H3′ interface that generate complementary binding surfaces [78]. An analogous approach was employed by the Zhou lab to generate a pair of H3 histones termed H3^D^ and H3^H^ with similar bump-hole mutations [79]. While these approaches are limited to histone H3, they eliminate the need for any additional processing steps after histone octamer assembly and yield almost exclusively asymmetric octamers. Crucially, these mutations can also be used to interrogate the consequences of nucleosomal asymmetry *in vivo*. In yeast, H3K36me3 is thought to silence transcription from cryptic internal promoters by recruiting the RPD3S histone deacetylase complex [80–82]. By blocking H3K36 methylation via introduction of a H3K36Q mutation in asymmetric fashion, a single H3K36 methylation site per nucleosome was shown to be sufficient to repress cryptic transcription in yeast [78,79]. Although application to mammalian systems is complicated by the presence of multiple genes encoding histone H3, these heterodimerization approaches should prove useful in studying the consequences of asymmetry *in vivo*.

Several methods have also been developed to generate hexasomes and heterotypic nucleosomes containing different H2A/H2B dimers [83,84]. These methods exploit asymmetry inherent in the 601 nucleosome positioning sequence, a synthetic DNA sequence with high affinity for histones that is routinely used for assembling recombinant nucleosomes *in vitro* [85]. First, a single H2A/H2B dimer can be assembled onto the 601 sequence in an oriented fashion to yield hexasomes. Subsequently, a different species of H2A/H2B dimers can be added to reconstitute asymmetric nucleosomes. The Bowman lab used this approach to study the mechanisms by which the chromatin remodeller Chd1 repositions nucleosomes, showing that it can utilize hexasomes as substrates and slide them in a unidirectional fashion [83]. The Narlikar lab used this approach to show that the yeast chromatin remodelling complex INO80 preferentially slides hexasomes in regulating nucleosome spacing [86]. The Muir lab developed these methods further, most recently by employing a truncated 601 sequence for the initial hexasome assembly, eliminating formation of nucleosome by-products. The missing part of the 601 sequence can then be ligated onto the truncated 601 DNA before a different type of dimer is added to form asymmetric nucleosomes [84]. This method can be combined with the spytethering approach described above [63] to assemble nucleosomes carrying asymmetric modifications on two different types of histones such as H3 and H2B.

Taken together, several approaches are now available for the generation of various types of asymmetric nucleosomes *in vitro*. These powerful tools will undoubtedly continue to facilitate the biochemical characterization of the mechanisms giving rise to asymmetry and their impact on the readout of histone marks, variants, and nucleosomal states. Despite these advances, however, it has remained challenging to study asymmetry in cells and organisms. While heterodimerization-based approaches such as the histone H3X/H3Y system allow enforcing asymmetry of specific marks, they rely on genetic mutations that prevent or facilitate a specific modification, thereby introducing non-native nucleosomes into chromatin. Moreover, these approaches currently lack the ability to modulate asymmetry in a locus-specific fashion. Controlling asymmetry by harnessing the potential of DNA sequence and base composition to differentially regulate histone modifier activity towards the two nucleosome halves represents an intriguing possibility. However, we currently lack sufficient mechanistic understanding to do so in a controlled and directed fashion, and changing DNA sequence would likely have additional unintended consequences beyond changing nucleosomal asymmetry. The development of methods to accurately control asymmetry in cells and to determine the genome-wide distribution of asymmetrically modified nucleosomes would greatly facilitate exploring the physiological roles of nucleosomal asymmetry.

## Functional consequences of nucleosomal asymmetry

Once established, nucleosomal asymmetry may regulate the interaction between chromatin binding proteins and nucleosomes, shaping chromatin structure and controlling chromatin-templated processes such as transcription. The symmetry state of a histone mark or variant changes its abundance and spatial arrangement at a given stretch of chromatin. By increasing the local concentration of available binding sites symmetric modification could enhance interaction with cognate reader domains. In addition, asymmetry controls which combinations of histone marks can be incorporated into a single nucleosome, affecting availability and relative orientation of different binding sites. In this way, asymmetry regulates multivalent interactions between nucleosomes and chromatin complexes that contain multiple reader domains for different histone marks, controlling affinity and specificity of their recruitment. In the case of dimeric reader proteins, symmetric modification may allow for simultaneous, multivalent engagement of the same mark on a single nucleosome, if such an interaction is sterically feasible. Crucially, symmetric modification is also required for dimeric readers to efficiently bridge neighboring nucleosomes. For instance, such a binding mode is thought to be key to the function of HP1 in mediating chromatin compaction and heterochromatin spreading [87,88].

Several studies have begun to explore how these consequences of asymmetry affect interactions in chromatin and regulate chromatin function. Thus far, these studies have primarily focused on transcription, bivalent chromatin, and oncohistone mutations in cancer.

### Roles of nucleosomal asymmetry in regulating transcription

By employing a high-resolution ChIP approach, the Pugh lab observed the presence of asymmetric nucleosomes around the TSS of around half of all genes in budding yeast [22]. This asymmetry manifests as increased levels of H3K9ac and H2BK123ub on the side of the +1 nucleosome that faces the NFR [22]. In contrast, the histone variant H2A.Z is enriched on the distal side [22], in line with the role of the NFR and adjacent nucleosomal DNA sequence in regulating SWR-mediated dimer exchange (see above). Moreover, histones were asymmetrically lost from the +1 nucleosome, leading to the formation of hexasomes and even half-nucleosomes [22]. This asymmetric nucleosome structure may promote transcription by facilitating passage of RNA polymerase II through the +1 nucleosome and its progression into gene bodies. In addition, the presence of hexasomes likely affects the activity of specific chromatin remodelers as highlighted above [83,86]. In mouse trophoblast stem cells, TSS of actively transcribed genes were also found to be marked by heterotypic H2A–H2A.Z nucleosomes [23], suggesting that the presence of such asymmetric nucleosomes may similarly facilitate transcription in mammals. RNA polymerase II travelling through the gene body in a directional fashion during elongation may impose further asymmetry beyond the TSS, resulting in asymmetric nucleosomes interacting with the transcription, chaperone, and chromatin remodeling machinery during transcription.

Promoters of active genes in mammals are marked by H3K4me3, which is thought to facilitate transcription by recruiting the basal transcription factor TFIID and other effectors such as HAT complexes and chromatin remodelers [56,58]. Using a calibrated ChIP-seq approach based on spike-in of internal standards that allows to obtain normalized histone modification densities that can be directly compared between different loci, the Ruthenburg lab observed that transcriptional output correlated with H3K4me3 levels at promoters in mouse ESCs [32]. Strikingly, in the resulting sigmoidal curve, mRNA abundance responded most strongly to increases in promoter H3K4me3 levels where they transition from asymmetric to those corresponding to symmetric nucleosomes. These findings suggest that the symmetry state of H3K4me3 may regulate recruitment of TFIID and other H3K4me3 readers by altering the availability and valence of binding sites for these readers and thus the avidity of their interaction with promoter nucleosomes. Supporting this notion, recent work from our lab showed that symmetric H3K4me3 modification allows efficient recruitment of TFIID and other H3K4me3 readers in nucleosome pulldown experiments, whereas asymmetric H3K4me3 failed to do so [89]. Together, these findings suggest that nucleosomal asymmetry of H3K4me3 and potentially other marks may regulate transcription by controlling the recruitment of histone mark readers.

### Nucleosomal asymmetry and bivalent domains

A direct consequence of nucleosomal asymmetry is the ability to generate patterns of combinatorial histone marks that would otherwise be prohibited. As a prominent example, the so-called bivalent domains adopt an asymmetric configuration, carrying H3K4me3 and H3K27me3 on separate H3 sister histones, thereby accommodating both marks within the same nucleosome. Mass spectrometry- and single molecule imaging-based studies conclusively showed that asymmetrically modified bivalent nucleosomes exist while symmetrically modified bivalent nucleosomes are essentially absent from mouse ESCs [20,21]. Although they cannot distinguish between asymmetric and symmetric nucleosomes, several re-ChIP-seq studies have confirmed the simultaneous presence of H3K4me3 and H3K27me3 at nucleosomes of bivalent promoters genome-wide [90–93]. Based on internal standard-calibrated ChIP-seq, promoters of developmentally regulated genes in mouse ESCs have a combined H3K4me3 and H3K27me3 modification density of ∼100% [32], consistent with asymmetric bivalent nucleosomes being the prevalent modification state at these promoters. In addition, engineered bivalent readers consisting of the H3K4me3-binding TAF3 PHD domain fused to the CBX7 or Drosophila Polycomb chromodomain, which bind H3K27me3, showed strong enrichment at bivalent promoters [94]. In contrast, engineered monovalent PHD-PHD and chromodomain-chromodomain readers exhibited poor enrichment at bivalent loci but were efficiently recruited to their cognate monovalent loci [94], arguing against presence of monovalent symmetric nucleosomes at bivalent promoters. Taken together, these studies indicate that bivalent promoters predominantly feature asymmetrically modified nucleosomes.

Once established, the asymmetric state of bivalent nucleosomes may regulate their function in poising developmental genes for expression. As a first layer, the inhibition of PRC2 by H3K4me3 on the same histone tail and the attenuation of SET1 and MLL1 activity by H3K27me3 provides a mechanism that prevents conversion of these promoters to a fully active or repressed state [67,95]. Upon differentiation, recruitment of transcription factors could skew the balance between activation and repression maintained at bivalent promoters, overriding this interplay to trigger activation. In addition, the asymmetric state may regulate the recruitment of effector proteins. As bivalency effectively enforces an asymmetric state for H3K4me3, recruitment of TFIID and other transcriptional activators may be diminished as discussed above, hindering activation of bivalent promoters. Moreover, asymmetric bivalent nucleosomes may recruit multivalent readers capable of interacting with both marks. Indeed, in Arabidopsis, the proteins EBS and SHL each contain both a PHD finger and a bromo adjacent homology (BAH) domain, which interact with H3K4me3 and H3K27me3, respectively [96–98]. While each protein can only bind either H3K4me3 via the PHD finger or H3K27me3 via the BAH domain but not both marks simultaneously, EBS and SHL homo- and heterodimerize to form bivalent reader complexes [98]. These complexes bind the bivalent state at the cold memory element region of the flowering control locus, driving cold-induced switching to a Polycomb repressed chromatin state [98]. Our recent work shows that factors capable of multivalent readout of the asymmetric bivalent state also exist in mammals [89]. Nucleosome binding of the HAT complex KAT6B is enhanced by the asymmetric bivalent modification, promoting recruitment of KAT6B to bivalent promoters in ESCs, where the complex is required for proper expression of developmental genes during differentiation [89]. Together, these studies suggest that asymmetry at bivalent promoters not only allows the bivalent methylation marks to coexist but also actively regulates their readout and function.

### Nucleosomal asymmetry and cancer

The discovery that several types of cancers are associated with specific somatic mutations in genes encoding core histones has spurred intense investigation into the mechanisms by which these mutations give rise to cancer phenotypes. Since the initial identification of the H3K27M and H3G34R/V mutations in pediatric gliomas, whole genome sequencing has revealed a plethora of histone mutations in a diverse range of cancers [25–29]. Given that these mutations only affect a single copy of several genes encoding the same histone, the resulting mutant histones only represent a small fraction of the total cellular histone pool. Oncohistones are therefore predominantly incorporated into heterotypic nucleosomes in chromatin, as has been experimentally verified in some cases (see e.g. [30,31]). The resulting asymmetry may contribute to the mechanisms by which oncohistones disrupt chromatin regulation. So far, the role of oncohistone asymmetry has mainly been studied for mutations affecting the nucleosome acidic patch. The acidic patch is a binding pocket formed by acidic residues of histones H2A and H2B, which is engaged by many chromatin factors in their multivalent interaction with nucleosomes [99], including many chromatin remodelers. In line with the importance of acidic patch-mediated interactions, cancer mutations in histones H2A and H2B have been shown to affect the activity of the ISWI family remodeler Snf2h *in vitro* [100,101]. Interestingly, when these mutations were incorporated into nucleosomes asymmetrically, they caused the remodeler to slide nucleosomes in a unidirectional rather than bidirectional fashion, leading to aberrant nucleosome spacing [101]. Directionality was similarly imposed on the activity of the cBAF chromatin remodeling complex [84]. These findings suggest that this group of oncohistones may modulate nucleosome positioning and chromatin accessibility, dysregulating transcription and gene expression profiles in cancer. Moreover, these studies underscore how nucleosomal asymmetry is an important regulator of chromatin states and gene expression.

Several cancers also exhibit overexpression of the histone H3 variant CENP-A [102]. CENP-A (also known as CenH3) defines the location of centromeres in most eukaryotes and serves as the platform for the assembly of kinetochores, which attach chromosomes to spindle microtubules during cell division to ensure accurate chromosome segregation [103]. While several lines of evidence suggest that CENP-A normally forms symmetric, homotypic nucleosomes at centromeres [104], overexpression of CENP-A in cancers can lead to its incorporation outside of centromeres in the form of heterotypic CENP-A–H3.3 nucleosomes [105]. In HeLa cells, such ectopic deposition has been shown to occur at active promoters and enhancers and CTCF binding sites [105]. Even though gene expression remained largely unchanged in this overexpression system, heterotypic CENP-A nucleosomes might deregulate gene expression in cancer by altering transcription factor binding and histone modification states. Moreover, heterotypic CENP-A nucleosomes at ectopic sites may also destabilise native centromeres by acting as a sink for centromere and kinetochore proteins, leading to chromosome segregation defects and aneuploidy [106].

## Concluding remarks

Since the discovery that nucleosomes can be asymmetrically modified, this novel facet of epigenetics and chromatin biology has attracted increasing attention. It is now well established that nucleosomal asymmetry is a hallmark of bivalent chromatin, and several lines of evidence suggest that asymmetry is a key regulator of transcription. So far, the roles of asymmetry have only been explored for a small subset of histone modification systems. Future studies will likely uncover additional regulatory systems and processes that rely on nucleosomal asymmetry. The development of novel methods to generate asymmetric nucleosomes has been pivotal to our ability to determine the mechanisms by which asymmetry is established, sets up complex histone modification patterns, and regulates multivalent interactions with chromatin reader complexes. Going forward, tools to map and quantify asymmetry in chromatin with nucleosome resolution as well as approaches to modulate and perturb asymmetry in a locus-specific and dynamic fashion in cells will be invaluable to gain a complete understanding of the physiological roles of nucleosomal asymmetry in regulating chromatin structure and function.

## Perspectives

- Despite their inherent symmetry, nucleosomes can carry different modifications on sister histones or contain different variants of the same core histone. The resulting nucleosomal asymmetry represents a novel layer of regulation in the establishment, readout, and function of chromatin states.
- Asymmetry expands the combinatorial space available to set up complex histone mark codes and regulates multivalent interactions between nucleosomes and chromatin complexes, thereby regulating processes such as transcription and the poising of developmental gene expression by bivalent chromatin.
- Furthering insight into the physiological role of nucleosomal asymmetry and the mechanisms by which it regulates chromatin structure and function requires the development of novel methods to generate asymmetric nucleosomes *in vitro*, map their localization and abundance in cells, and to alter asymmetry in a locus-specific fashion to examine how asymmetry regulates transcription and other processes.

## Abbreviations

BAH: bromo adjacent homology
ChIP: chromatin immunoprecipitation
CHK1: checkpoint kinase 1
ESC: embryonic stem cells
HAT: histone acetyltransferase
NFR: nucleosome free region
PKC: protein kinase C
PRC2: Polycomb repressive complex 2
TSS: transcriptional start site

## References

[1] Luger, K., Mäder, A.W., Richmond, R.K., Sargent, D.F. and Richmond, T.J. (1997) Crystal structure of the nucleosome core particle at 2.8 A resolution. Nature 389, 251–260 10.1038/38444 9305837

[2] Kornberg, R.D. and Lorch, Y. (1999) Twenty-five years of the nucleosome, fundamental particle of the eukaryote chromosome. Cell 98, 285–294 10.1016/S0092-8674(00)81958-3 10458604

[3] Millán-Zambrano, G., Burton, A., Bannister, A.J. and Schneider, R. (2022) Histone post-translational modifications — cause and consequence of genome function. Nat. Rev. Genet. 23, 563–580 10.1038/s41576-022-00468-7 35338361

[4] Bannister, A.J. and Kouzarides, T. (2011) Regulation of chromatin by histone modifications. Cell Res. 21, 381–395 10.1038/cr.2011.22 21321607 PMC3193420

[5] Martire, S. and Banaszynski, L.A. (2020) The roles of histone variants in fine-tuning chromatin organization and function. Nat. Rev. Mol. Cell Biol. 21, 522–541 10.1038/s41580-020-0262-8 32665685 PMC8245300

[6] Buschbeck, M. and Hake, S.B. (2017) Variants of core histones and their roles in cell fate decisions, development and cancer. Nat. Rev. Mol. Cell Biol. 18, 299–314 10.1038/nrm.2016.166 28144029

[7] Allis, C.D. and Jenuwein, T. (2016) The molecular hallmarks of epigenetic control. Nat. Rev. Genet. 17, 487–500 10.1038/nrg.2016.59 27346641

[8] Li, B., Carey, M. and Workman, J.L. (2007) The role of chromatin during transcription. Cell 128, 707–719 10.1016/j.cell.2007.01.015 17320508

[9] Jenuwein, T. and Allis, C.D. (2001) Translating the histone code. Science 293, 1074–1080 10.1126/science.1063127 11498575

[10] Strahl, B.D. and Allis, C.D. (2000) The language of covalent histone modifications. Nature 403, 41–45 10.1038/47412 10638745

[11] Mikkelsen, T.S., Ku, M., Jaffe, D.B., Issac, B., Lieberman, E., Giannoukos, G. et al. (2007) Genome-wide maps of chromatin state in pluripotent and lineage-committed cells. Nature 448, 553–560 10.1038/nature06008 17603471 PMC2921165

[12] Zhou, V.W., Goren, A. and Bernstein, B.E. (2011) Charting histone modifications and the functional organization of mammalian genomes. Nat. Rev. Genet. 12, 7–18 10.1038/nrg2905 21116306

[13] Lee, J.-S., Smith, E. and Shilatifard, A. (2010) The language of histone crosstalk. Cell 142, 682–685 10.1016/j.cell.2010.08.011 20813257 PMC3711869

[14] Suganuma, T. and Workman, J.L. (2008) Crosstalk among histone modifications. Cell 135, 604–607 10.1016/j.cell.2008.10.036 19013272

[15] Zhang, T., Cooper, S. and Brockdorff, N. (2015) The interplay of histone modifications - writers that read. EMBO Rep. 16, 1467–1481 10.15252/embr.201540945 26474904 PMC4641500

[16] Wang, Z. and Patel, D.J. (2011) Combinatorial readout of dual histone modifications by paired chromatin-associated modules. J. Biol. Chem. 286, 18363–18368 10.1074/jbc.R111.219139 21454653 PMC3099652

[17] Ruthenburg, A.J., Li, H., Patel, D.J. and Allis, C.D. (2007) Multivalent engagement of chromatin modifications by linked binding modules. Nat. Rev. Mol. Cell Biol. 8, 983–994 10.1038/nrm2298 18037899 PMC4690530

[18] Su, Z. and Denu, J.M. (2016) Reading the combinatorial histone language. ACS Chem. Biol. 11, 564–574 10.1021/acschembio.5b00864 26675328 PMC10127456

[19] Musselman, C.A., Lalonde, M.-E., Côté, J. and Kutateladze, T.G. (2012) Perceiving the epigenetic landscape through histone readers. Nat. Struct. Mol. Biol 19, 1218–1227 10.1038/nsmb.2436 23211769 PMC3645987

[20] Shema, E., Jones, D., Shoresh, N., Donohue, L., Ram, O. and Bernstein, B.E. (2016) Single-molecule decoding of combinatorially modified nucleosomes. Science 352, 717–721 10.1126/science.aad7701 27151869 PMC4904710

[21] Voigt, P., Leroy, G., Iii, W.J.D., Zee, B.M., Son, J., Beck, D.B. et al. (2012) Asymmetrically modified nucleosomes. Cell 151, 181–193 10.1016/j.cell.2012.09.002 23021224 PMC3498816

[22] Rhee, H.S., Bataille, A.R., Zhang, L. and Pugh, B.F. (2014) Subnucleosomal structures and nucleosome asymmetry across a genome. Cell 159, 1377–1388 10.1016/j.cell.2014.10.054 25480300 PMC4258235

[23] Nekrasov, M., Amrichova, J., Parker, B.J., Soboleva, T.A., Jack, C., Williams, R. et al. (2012) Histone H2A.Z inheritance during the cell cycle and its impact on promoter organization and dynamics. Nat. Struct. Mol. Biol. 19, 1076–1083 10.1038/nsmb.2424 23085713

[24] Weber, C.M., Henikoff, J.G. and Henikoff, S. (2010) H2a.Z nucleosomes enriched over active genes are homotypic. Nat. Struct. Mol. Biol. 17, 1500–1507 10.1038/nsmb.1926 21057526 PMC3051840

[25] Deshmukh, S., Ptack, A., Krug, B. and Jabado, N. (2022) Oncohistones: a roadmap to stalled development. FEBS J. 289, 1315–1328 10.1111/febs.15963 33969633 PMC9990449

[26] Sturm, D., Bender, S., Jones, D.T.W., Lichter, P., Grill, J., Becher, O. et al. (2014) Paediatric and adult glioblastoma: multiform (epi)genomic culprits emerge. Nat. Rev. Cancer 14, 92–107 10.1038/nrc3655 24457416 PMC4003223

[27] Mitchener, M.M. and Muir, T.W. (2022) Oncohistones: exposing the nuances and vulnerabilities of epigenetic regulation. Mol. Cell 82, 2925–2938 10.1016/j.molcel.2022.07.008 35985302 PMC9482148

[28] Nacev, B.A., Feng, L., Bagert, J.D., Lemiesz, A.E., Gao, J., Soshnev, A.A. et al. (2019) The expanding landscape of ‘oncohistone’ mutations in human cancers. Nature 567, 473–478 10.1038/s41586-019-1038-1 30894748 PMC6512987

[29] Pereira, K.N.E., Shan, J., Licht, J.D. and Bennett, R.L. (2023) Histone mutations in cancer. Biochem. Soc. Trans. 51, 1749–1763 10.1042/BST20210567 PMC10657182 37721138

[30] Lewis, P.W., Müller, M.M., Koletsky, M.S., Cordero, F., Lin, S., Banaszynski, L.A. et al. (2013). Inhibition of PRC2 activity by a gain-of-function H3 mutation found in pediatric glioblastoma. Science 340, 857–861 10.1126/science.1232245 23539183 PMC3951439

[31] Piunti, A., Hashizume, R., Morgan, M.A., Bartom, E.T., Horbinski, C.M., Marshall, S.A. et al. (2017) Therapeutic targeting of polycomb and BET bromodomain proteins in diffuse intrinsic pontine gliomas. Nat. Med. 23, 493–500 10.1038/nm.4296 28263307 PMC5667640

[32] Grzybowski, A.T., Chen, Z. and Ruthenburg, A.J. (2015) Calibrating ChIP-seq with nucleosomal internal standards to measure histone modification density genome wide. Mol. Cell 58, 886–899 10.1016/j.molcel.2015.04.022 26004229 PMC4458216

[33] Liokatis, S., Stützer, A., Elsässer, S.J., Theillet, F.-X., Klingberg, R., Rossum, B.V. et al. (2012) Phosphorylation of histone H3 Ser10 establishes a hierarchy for subsequent intramolecular modification events. Nat. Struct. Mol. Biol. 19, 819–823 10.1038/nsmb.2310 22796964

[34] Weinert, B.T., Narita, T., Satpathy, S., Srinivasan, B., Hansen, B.K., Schölz, C. et al. (2018) Time-resolved analysis reveals rapid dynamics and broad scope of the CBP/p300 acetylome. Cell 174, 231–244.e212 10.1016/j.cell.2018.04.033 29804834 PMC6078418

[35] Clapier, C.R., Iwasa, J., Cairns, B.R. and Peterson, C.L. (2017) Mechanisms of action and regulation of ATP-dependent chromatin-remodelling complexes. Nat. Rev. Mol. Cell Biol. 18, 407–422 10.1038/nrm.2017.26 28512350 PMC8127953

[36] Singh, R.K., Fan, J., Gioacchini, N., Watanabe, S., Bilsel, O. and Peterson, C.L. (2019) Transient kinetic analysis of SWR1C-catalyzed H2A.Z deposition unravels the impact of nucleosome dynamics and the asymmetry of histone exchange. Cell Rep. 27, 374–386.e374 10.1016/j.celrep.2019.03.035 30970243 PMC6545893

[37] Ranjan, A., Mizuguchi, G., FitzGerald, P.C., Wei, D., Wang, F., Huang, Y. et al. (2013) Nucleosome-free region dominates histone acetylation in targeting SWR1 to promoters for H2A.Z replacement. Cell 154, 1232–1245 10.1016/j.cell.2013.08.005 24034247 PMC3815578

[38] Sun, L., Pierrakeas, L., Li, T. and Luk, E. (2020) Thermosensitive nucleosome editing reveals the role of DNA sequence in targeted histone variant deposition. Cell Rep. 30, 257–268.e255 10.1016/j.celrep.2019.12.006 31914392 PMC7041490

[39] García, A., Durán, L., Sánchez, M., González, S., Santamaría, R. and Antequera, F. (2024) Asymmetrical nucleosomal DNA signatures regulate transcriptional directionality. Cell Rep. 43, 113605 10.1016/j.celrep.2023.113605 38127622

[40] Winger, J. and Bowman, G.D. (2017) The sequence of nucleosomal DNA modulates sliding by the Chd1 chromatin remodeler. J. Mol. Biol. 429, 808–822 10.1016/j.jmb.2017.02.002 28189426 PMC5357180

[41] Basu, A., Bobrovnikov, D.G., Qureshi, Z., Kayikcioglu, T., Ngo, T.T.M., Ranjan, A. et al. (2021) Measuring DNA mechanics on the genome scale. Nature 589, 462–467 10.1038/s41586-020-03052-3 33328628 PMC7855230

[42] Basu, A., Bobrovnikov, D.G., Cieza, B., Arcon, J.P., Qureshi, Z., Orozco, M. et al. (2022) Deciphering the mechanical code of the genome and epigenome. Nat. Struct. Mol. Biol. 29, 1178–1187 10.1038/s41594-022-00877-6 36471057 PMC10142808

[43] Mauney, A.W., Tokuda, J.M., Gloss, L.M., Gonzalez, O. and Pollack, L. (2018) Local DNA sequence controls asymmetry of DNA unwrapping from nucleosome core particles. Biophys. J. 115, 773–781 10.1016/j.bpj.2018.07.009 30072033 PMC6127449

[44] Ngo, T.T.M., Zhang, Q., Zhou, R., Yodh, J.G. and Ha, T. (2015) Asymmetric unwrapping of nucleosomes under tension directed by DNA local flexibility. Cell 160, 1135–1144 10.1016/j.cell.2015.02.001 25768909 PMC4409768

[45] Albaugh, B.N. and Denu, J.M. (2021) Catalysis by protein acetyltransferase Gcn5. Biochim. Biophys. Acta (BBA) - Gene Regul. Mech. 1864, 194627 10.1016/j.bbagrm.2020.194627 PMC7854473 32841743

[46] Strahl, B.D. and Briggs, S.D. (2021) The SAGA continues: the rise of cis- and trans-histone crosstalk pathways. Biochim. Biophys. Acta (BBA) - Gene Regul. Mech. 1864, 194600 10.1016/j.bbagrm.2020.194600 PMC7785665 32645359

[47] Li, S. and Shogren-Knaak, M.A. (2009) The Gcn5 bromodomain of the SAGA complex facilitates cooperative and cross-tail acetylation of nucleosomes. J. Biol. Chem. 284, 9411–9417 10.1074/jbc.M809617200 19218239 PMC2666593

[48] Li, S. and Shogren-Knaak, M.A. (2008) Cross-talk between histone H3 tails produces cooperative nucleosome acetylation. Proc. Natl Acad. Sci. U.S.A. 105, 18243–18248 10.1073/pnas.0804530105 19004784 PMC2587550

[49] Blackledge, N.P. and Klose, R.J. (2021) The molecular principles of gene regulation by Polycomb repressive complexes. Nat. Rev. Mol. Cell Biol. 22, 815–833 10.1038/s41580-021-00398-y 34400841 PMC7612013

[50] Yu, J.-R., Lee, C.-H., Oksuz, O., Stafford, J.M. and Reinberg, D. (2019) PRC2 is high maintenance. Genes Dev. 33, 903–935 10.1101/gad.325050.119 31123062 PMC6672058

[51] Piunti, A. and Shilatifard, A. (2021) The roles of Polycomb repressive complexes in mammalian development and cancer. Nat. Rev. Mol. Cell Biol. 22, 326–345 10.1038/s41580-021-00341-1 33723438

[52] Margueron, R., Justin, N., Ohno, K., Sharpe, M.L., Son, J., Drury, W.J. et al. (2009) Role of the polycomb protein EED in the propagation of repressive histone marks. Nature 461, 762–767 10.1038/nature08398 19767730 PMC3772642

[53] Lechner, C.C., Agashe, N.D. and Fierz, B. (2016) Traceless synthesis of asymmetrically modified bivalent nucleosomes. Angew. Chem. Int. Ed. Engl. 55, 2903–2906 10.1002/anie.201510996 26806951

[54] Guidotti, N., Lechner, C.C., Bachmann, A.L. and Fierz, B. (2019) A modular ligation strategy for asymmetric bivalent nucleosomes trimethylated at K36 and K27. ChemBioChem 20, 1124–1128 10.1002/cbic.201800744 30615245

[55] Shilatifard, A. (2012) The COMPASS family of histone H3K4 methylases: mechanisms of regulation in development and disease pathogenesis. Annu. Rev. Biochem. 81, 65–95 10.1146/annurev-biochem-051710-134100 22663077 PMC4010150

[56] Hughes, A.L., Kelley, J.R. and Klose, R.J. (2020) Understanding the interplay between CpG island-associated gene promoters and H3K4 methylation. Biochim. Biophys. Acta (BBA) - Gene Regul. Mech. 1863, 194567 10.1016/j.bbagrm.2020.194567 PMC7294231 32360393

[57] Shi, X., Kachirskaia, I., Walter, K.L., Kuo, J.-H.A., Lake, A., Davrazou, F. et al. (2007) Proteome-wide analysis in *Saccharomyces cerevisiae* identifies several PHD fingers as novel direct and selective binding modules of histone H3 methylated at either lysine 4 or lysine 36. J. Biol. Chem. 282, 2450–2455 10.1074/jbc.C600286200 17142463 PMC2735445

[58] Eberl, H.C., Spruijt, C.G., Kelstrup, C.D., Vermeulen, M. and Mann, M. (2013) A map of general and specialized chromatin readers in mouse tissues generated by label-free interaction proteomics. Mol. Cell 49, 368–378 10.1016/j.molcel.2012.10.026 23201125

[59] Choudhury, R., Singh, S., Arumugam, S., Roguev, A. and Stewart, A.F. (2019) The Set1 complex is dimeric and acts with Jhd2 demethylation to convey symmetrical H3K4 trimethylation. Genes Dev. 33, 550–564 10.1101/gad.322222.118 30842216 PMC6499330

[60] Torres, I.O., Kuchenbecker, K.M., Nnadi, C.I., Fletterick, R.J., Kelly, M.J.S. and Fujimori, D.G. (2015) Histone demethylase KDM5A is regulated by its reader domain through a positive-feedback mechanism. Nat. Commun. 6, 6204 10.1038/ncomms7204 25686748 PMC5080983

[61] Klein, B.J., Piao, L., Xi, Y., Rincon-Arano, H., Rothbart, S.B., Peng, D. et al. (2014) The histone-H3K4-specific demethylase KDM5B binds to its substrate and product through distinct PHD fingers. Cell Rep. 6, 325–335 10.1016/j.celrep.2013.12.021 24412361 PMC3918441

[62] Zhang, Y., Yang, H., Guo, X., Rong, N., Song, Y., Xu, Y. et al. (2014) The PHD1 finger of KDM5B recognizes unmodified H3K4 during the demethylation of histone H3K4me2/3 by KDM5B. Protein Cell 5, 837–850 10.1007/s13238-014-0078-4 24952722 PMC4225485

[63] Lukasak, B.J., Thompson, R.E., Mitchener, M.M., Feng, V.J., Bagert, J.D. and Muir, T.W. (2022) A genetically encoded approach for breaking chromatin symmetry. ACS Cent. Sci. 8, 176–183 10.1021/acscentsci.1c01332 35233450 PMC8875426

[64] Margueron, R., Li, G., Sarma, K., Blais, A., Zavadil, J., Woodcock, C.L. et al. (2008) Ezh1 and Ezh2 maintain repressive chromatin through different mechanisms. Mol. Cell 32, 503–518 10.1016/j.molcel.2008.11.004 19026781 PMC3641558

[65] Son, J., Shen, S.S., Margueron, R. and Reinberg, D. (2013) Nucleosome-binding activities within JARID2 and EZH1 regulate the function of PRC2 on chromatin. Genes Dev. 27, 2663–2677 10.1101/gad.225888.113 24352422 PMC3877756

[66] Davidovich, C., Goodrich, K.J., Gooding, A.R. and Cech, T.R. (2014) A dimeric state for PRC2. Nucleic Acids Res. 42, 9236–9248 10.1093/nar/gku540 24992961 PMC4132707

[67] Schmitges, F.W., Prusty, A.B., Faty, M., Stützer, A., Lingaraju, G.M., Aiwazian, J. et al. (2011) Histone methylation by PRC2 is inhibited by active chromatin marks. Mol. Cell 42, 330–341 10.1016/j.molcel.2011.03.025 21549310

[68] Voigt, P., Tee, W.-W. and Reinberg, D. (2013) A double take on bivalent promoters. Genes Dev. 27, 1318–1338 10.1101/gad.219626.113 23788621 PMC3701188

[69] Azuara, V., Perry, P., Sauer, S., Spivakov, M., Jørgensen, H.F., John, R.M. et al. (2006) Chromatin signatures of pluripotent cell lines. Nat. Cell Biol. 8, 532–538 10.1038/ncb1403 16570078

[70] Bernstein, B.E., Mikkelsen, T.S., Xie, X., Kamal, M., Huebert, D.J., Cuff, J. et al. (2006) A bivalent chromatin structure marks key developmental genes in embryonic stem cells. Cell 125, 315–326 10.1016/j.cell.2006.02.041 16630819

[71] Macrae, T.A., Fothergill-Robinson, J. and Ramalho-Santos, M. (2023) Regulation, functions and transmission of bivalent chromatin during mammalian development. Nat. Rev. Mol. Cell Biol. 24, 6–26 10.1038/s41580-022-00518-2 36028557

[72] Blanco, E., González-Ramírez, M., Alcaine-Colet, A., Aranda, S. and Croce, L.D. (2020) The bivalent genome: characterization, structure, and regulation. Trends Genet. 36, 118–131 10.1016/j.tig.2019.11.004 31818514

[73] Liokatis, S., Klingberg, R., Tan, S. and Schwarzer, D. (2016) Differentially isotope-labeled nucleosomes to study asymmetric histone modification crosstalk by time-resolved NMR spectroscopy. Angew. Chem. Int. Ed. Engl. 55, 8262–8265 10.1002/anie.201601938 27219518 PMC4939407

[74] Mitchener, M.M. and Muir, T.W. (2021) Janus bioparticles: asymmetric nucleosomes and their preparation using chemical biology approaches. Acc. Chem. Res. 54, 3215–3227 10.1021/acs.accounts.1c00313 34319695 PMC8411803

[75] Voigt, P. and Reinberg, D. (2011) Histone tails: ideal motifs for probing epigenetics through chemical biology approaches. ChemBioChem 12, 236–252 10.1002/cbic.201000493 21243712 PMC3760146

[76] Hananya, N., Koren, S. and Muir, T.W. (2023) Interrogating epigenetic mechanisms with chemically customized chromatin. Nat. Rev. Genet. 25, 255–271 10.1038/s41576-023-00664-z 37985791 PMC11176933

[77] Müller, M.M. and Muir, T.W. (2015) Histones: at the crossroads of peptide and protein chemistry. Chem. Rev. 115, 2296–2349 10.1021/cr5003529 25330018 PMC4378460

[78] Ichikawa, Y., Connelly, C.F., Appleboim, A., Miller, T.C., Jacobi, H., Abshiru, N.A. et al. (2017) A synthetic biology approach to probing nucleosome symmetry. eLife 6, 1002 10.7554/eLife.28836 PMC5626479 28895528

[79] Zhou, Z., Liu, Y.-T., Ma, L., Gong, T., Hu, Y.-N., Li, H.-T. et al. (2017) Independent manipulation of histone H3 modifications in individual nucleosomes reveals the contributions of sister histones to transcription. eLife 6, e30178 10.7554/eLife.30178 29027902 PMC5677365

[80] Carrozza, M.J., Li, B., Florens, L., Suganuma, T., Swanson, S.K., Lee, K.K. et al. (2005) Histone H3 methylation by Set2 directs deacetylation of coding regions by Rpd3S to suppress spurious intragenic transcription. Cell 123, 581–592 10.1016/j.cell.2005.10.023 16286007

[81] Joshi, A.A. and Struhl, K. (2005) Eaf3 chromodomain interaction with methylated H3-K36 links histone deacetylation to Pol II elongation. Mol. Cell 20, 971–978 10.1016/j.molcel.2005.11.021 16364921

[82] Keogh, M.-C., Kurdistani, S.K., Morris, S.A., Ahn, S.H., Podolny, V., Collins, S.R. et al. (2005) Cotranscriptional Set2 methylation of histone H3 lysine 36 recruits a repressive Rpd3 complex. Cell 123, 593–605 10.1016/j.cell.2005.10.025 16286008

[83] Levendosky, R.F., Sabantsev, A., Deindl, S. and Bowman, G.D. (2016) The Chd1 chromatin remodeler shifts hexasomes unidirectionally. eLife 5, e21356 10.7554/eLife.21356 28032848 PMC5226652

[84] Dao, H.T., Liu, H., Mashtalir, N., Kadoch, C. and Muir, T.W. (2022) Synthesis of oriented hexasomes and asymmetric nucleosomes using a template editing process. J. Am. Chem. Soc. 144, 2284–2291 10.1021/jacs.1c12420 35081309 PMC8935522

[85] Lowary, P.T. and Widom, J. (1998) New DNA sequence rules for high affinity binding to histone octamer and sequence-directed nucleosome positioning. J. Mol. Biol. 276, 19–42 10.1006/jmbi.1997.1494 9514715

[86] Hsieh, L.J., Gourdet, M.A., Moore, C.M., Muñoz, E.N., Gamarra, N., Ramani, V. et al. (2022) A hexasome is the preferred substrate for the INO80 chromatin remodeling complex, allowing versatility of function. Mol. Cell 82, 2098–2112.e2094 10.1016/j.molcel.2022.04.026 35597239 PMC9351570

[87] Canzio, D., Chang, E.Y., Shankar, S., Kuchenbecker, K.M., Simon, M.D., Madhani, H.D. et al. (2011) Chromodomain-mediated oligomerization of HP1 suggests a nucleosome-bridging mechanism for heterochromatin assembly. Mol. Cell 41, 67–81 10.1016/j.molcel.2010.12.016 21211724 PMC3752404

[88] Canzio, D., Liao, M., Naber, N., Pate, E., Larson, A., Wu, S. et al. (2013) A conformational switch in HP1 releases auto-inhibition to drive heterochromatin assembly. Nature 496, 377–381 10.1038/nature12032 23485968 PMC3907283

[89] Bryan, E., Warburton, M., Webb, K.M., McLaughlin, K.A., Spanos, C., Ambrosi, C. et al. (2021) Nucleosomal asymmetry shapes histone mark binding and promotes poising at bivalent domains. bioRxiv 10.1101/2021.02.08.430127

[90] Kinkley, S., Helmuth, J., Polansky, J.K., Dunkel, I., Gasparoni, G., Fröhler, S. et al. (2016) reChIP-seq reveals widespread bivalency of H3K4me3 and H3K27me3 in CD4(+) memory T cells. Nat. Commun. 7, 12514 10.1038/ncomms12514 27530917 PMC4992058

[91] Mas, G., Blanco, E., Ballaré, C., Sansó, M., Spill, Y.G., Hu, D. et al. (2018) Promoter bivalency favors an open chromatin architecture in embryonic stem cells. Nat. Genet. 50, 1452–1462 10.1038/s41588-018-0218-5 30224650

[92] Sen, S., Block, K.F., Pasini, A., Baylin, S.B. and Easwaran, H. (2016) Genome-wide positioning of bivalent mononucleosomes. BMC Med. Genomics 9, 60 10.1186/s12920-016-0221-6 27634286 PMC5025636

[93] Weiner, A., Lara-Astiaso, D., Krupalnik, V., Gafni, O., David, E., Winter, D.R. et al. (2016) Co-ChIP enables genome-wide mapping of histone mark co-occurrence at single-molecule resolution. Nat. Biotechnol. 34, 953–961 10.1038/nbt.3652 27454738

[94] Villaseñor, R., Pfaendler, R., Ambrosi, C., Butz, S., Giuliani, S., Bryan, E. et al. (2020) ChromID identifies the protein interactome at chromatin marks. Nat. Biotechnol. 38, 728–736 10.1038/s41587-020-0434-2 32123383 PMC7289633

[95] Kim, D.-H., Tang, Z., Shimada, M., Fierz, B., Houck-Loomis, B., Bar-Dagen, M. et al. (2013) Histone H3K27 trimethylation inhibits H3 binding and function of SET1-like H3K4 methyltransferase complexes. Mol. Cell. Biol. 33, 4936–4946 10.1128/MCB.00601-13 24126056 PMC3889540

[96] Yang, Z., Qian, S., Scheid, R.N., Lu, L., Chen, X., Liu, R. et al. (2018) EBS is a bivalent histone reader that regulates floral phase transition in Arabidopsis. Nat. Genet. 50, 1247–1253 10.1038/s41588-018-0187-8 30082787 PMC6556434

[97] Qian, S., Lv, X., Scheid, R.N., Lu, L., Yang, Z., Chen, W. et al. (2018) Dual recognition of H3K4me3 and H3K27me3 by a plant histone reader SHL. Nat. Commun. 9, 2425–2411 10.1038/s41467-018-04836-y 29930355 PMC6013494

[98] Gao, Z., Li, Y., Ou, Y., Yin, M., Chen, T., Zeng, X. et al. (2023) A pair of readers of bivalent chromatin mediate formation of Polycomb-based “memory of cold” in plants. Mol. Cell 83, 1109–1124.e4 10.1016/j.molcel.2023.02.014 36921607

[99] McGinty, R.K. and Tan, S. (2021) Principles of nucleosome recognition by chromatin factors and enzymes. Curr. Opin. Struct. Biol. 71, 16–26 10.1016/j.sbi.2021.05.006 34198054 PMC8648869

[100] Bagert, J.D., Mitchener, M.M., Patriotis, A.L., Dul, B.E., Wojcik, F., Nacev, B.A. et al. (2021) Oncohistone mutations enhance chromatin remodeling and alter cell fates. Nat. Chem. Biol. 17, 403–411 10.1038/s41589-021-00738-1 33649601 PMC8174649

[101] Dao, H.T., Dul, B.E., Dann, G.P., Liszczak, G.P. and Muir, T.W. (2020) A basic motif anchoring ISWI to nucleosome acidic patch regulates nucleosome spacing. Nat. Chem. Biol. 16, 134–142 10.1038/s41589-019-0413-4 31819269 PMC6982587

[102] Sharma, A.B., Dimitrov, S., Hamiche, A. and Van Dyck, E. (2018) Centromeric and ectopic assembly of CENP-A chromatin in health and cancer: old marks and new tracks. Nucleic Acids Res. 47, 1051–1069 10.1093/nar/gky1298 PMC6379705 30590707

[103] McKinley, K.L. and Cheeseman, I.M. (2016) The molecular basis for centromere identity and function. Nat. Rev. Mol. Cell Biol. 17, 16–29 10.1038/nrm.2015.5 26601620 PMC8603311

[104] Dunleavy, E.M., Zhang, W. and Karpen, G.H. (2013) Solo or doppio: how many CENP-As make a centromeric nucleosome? Nat. Struct. Mol. Biol. 20, 648–650 10.1038/nsmb.2602 23739165

[105] Lacoste, N., Woolfe, A., Tachiwana, H., Garea, A.V., Barth, T., Cantaloube, S. et al. (2014) Mislocalization of the centromeric histone variant CenH3/CENP-A in human cells depends on the chaperone DAXX. Mol. Cell 53, 631–644 10.1016/j.molcel.2014.01.018 24530302

[106] Shrestha, R.L., Ahn, G.S., Staples, M.I., Sathyan, K.M., Karpova, T.S., Foltz, D.R. et al. (2017) Mislocalization of centromeric histone H3 variant CENP-A contributes to chromosomal instability (CIN) in human cells. Oncotarget 8, 46781–46800 10.18632/oncotarget.18108 28596481 PMC5564523

